# Stereotactic guidance for ventricular catheter placement in ventriculoperitoneal shunt surgery: a state-of-the-art review on technical rationale, clinical outcomes, and future directions

**DOI:** 10.3389/fneur.2026.1931318

**Published:** 2026-10-07

**Authors:** Erich Talamoni Fonoff, Igor Baroni Cardoso, Alexandre Maria Santos

**Affiliations:** 1 Instituto Parkinson Hoje, São Paulo, Brazil; 2 Department of Neurology, Discipline of Neurosurgery, University of São Paulo Medical School, University of São Paulo, São Paulo, Brazil; 3 Department of Health Sciences, Federal University of Paraná, Curitiba, Brazil

**Keywords:** catheter accuracy, cost-effectiveness, hemorrhage, hydrocephalus, neuronavigation, proximal shunt failure, stereotactic surgery, ventriculoperitoneal shunt

## Abstract

Ventriculoperitoneal (VP) shunting remains the most frequently performed surgical treatment for hydrocephalus; however, shunt failure rates remain substantial despite technical refinements. Proximal catheter malfunction, often associated with malposition, ventricular wall contact, or choroid plexus obstruction, represents a common cause of shunt revision. Conventional freehand ventricular cannulation is based on surface anatomical landmarks and surgical experience, which may be unreliable in patients with small or distorted ventricles. Stereotactic guidance—including frame-based systems, frameless neuronavigation, and electromagnetic navigation—has been introduced to improve placement accuracy and reproducibility. Randomized and cohort studies consistently demonstrate improved radiographic accuracy and higher first-pass success rates with guided techniques. However, systematic reviews have not uniformly shown reductions in overall shunt revision rates, likely reflecting the multifactorial etiology of shunt failure. Hemorrhagic complications after VP shunt placement are uncommon but clinically important; evidence from VP shunt cohorts and from the broader stereotactic neurosurgery experience (notably deep brain stimulation) suggests that trajectory planning and vascular/sulcal avoidance may reduce bleeding risk. This review synthesizes epidemiological context, technical rationale, comparative outcomes, hemorrhage considerations, health economic implications, and future research priorities regarding stereotactic ventricular catheter placement.

## Graphical abstract

**Figure:**
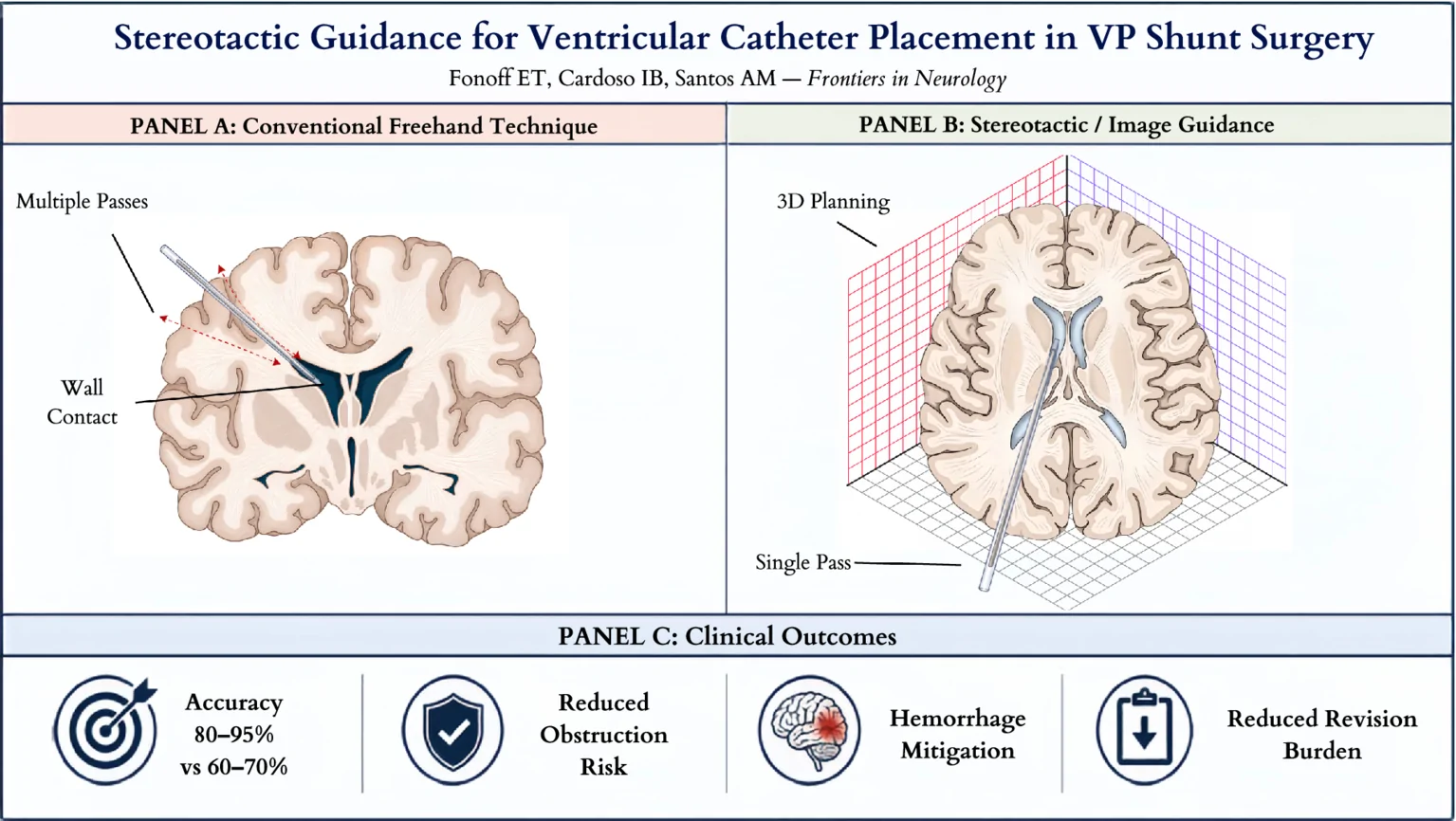
Stereotactic Guidance for Ventricular Catheter Placement in VP Shunt Surgery. Comparison of conventional freehand catheter placement (Panel A), which frequently requires multiple passes and carries a high risk of ventricular wall contact, against stereotactic and image-guided cannulation utilizing 3D trajectory planning to ensure a precise single pass (Panel B). As summarized in the clinical outcomes (Panel C), adopting stereotactic guidance increases catheter placement accuracy to 80–95% (compared to 60–70% with freehand methods), decreases the risk of catheter obstruction, mitigates parenchymal hemorrhage, and significantly reduces the long-term revision burden.

## Introduction

Hydrocephalus affects patients across the lifespan and represents a major source of neurosurgical morbidity worldwide (1, 2). Ventriculoperitoneal shunting remains the most widely employed treatment modality for both pediatric and adult hydrocephalus (3). Despite advances in valve technology and perioperative protocols, shunt failure rates remain high. First-year revision rates frequently fall in the 20–40% range in pediatric cohorts, and remain clinically significant in adults (4, 5).

Proximal catheter obstruction constitutes one of the most common causes of shunt malfunction (4–6). Mechanical occlusion may result from choroid plexus ingrowth, ependymal tissue proliferation, inflammatory debris, and/or suboptimal catheter positioning (6, 7). Radiographic analyses demonstrate that catheter malposition correlates with increased proximal revision risk, supporting the rationale that improving placement accuracy could improve proximal durability (8, 9).

Traditional freehand ventricular catheter placement relies on surface landmarks such as Kocher’s point and estimated trajectory toward the frontal horn. While effective in enlarged ventricles, accuracy declines in patients with small ventricles, slit ventricle syndrome, tumor-related distortion, midline shift, or revision shunt surgery (8–11). Variability in entry point, depth estimation, and trajectory may contribute to shunt failure through multiple mechanisms, such as ventricular wall contact and intraparenchymal fenestration placement.

Stereotactic and image-guided techniques, including frame-based stereotaxy, frameless neuronavigation, electromagnetic tracking systems, and ultrasound guidance, have been applied to shunt surgery to enhance precision and reproducibility (8–14). Their role in routine shunt placement remains debated, largely because the clinical endpoint of “overall revision” is influenced by infection, distal failure, and valve issues, which may be independent of proximal catheter accurate positioning (4, 5, 13). The primary objectives of this review are: (1) to evaluate whether the enhanced anatomical accuracy of stereotactic ventricular catheter placement translates into superior long-term proximal durability across specific patient subgroups (e.g., small ventricles, revision surgery); (2) to synthesize the pathophysiological mechanisms linking repeated cannulation attempts to catheter tract hemorrhage and proximal occlusion; and (3) to translate trajectory planning and sulcal avoidance principles from deep brain stimulation (DBS) surgery to hydrocephalus management.

### Literature search strategy

This study was designed and conducted as a structured, critical state-of-the-art review. To ensure methodological rigor, transparency, and high-quality reporting in narrative syntheses, our literature search, evaluation, and data presentation adhered to the principles of the Scale for the Assessment of Narrative Review Articles (SANRA) guidelines (15).

Literature search was conducted through PubMed/MEDLINE and Embase databanks, and sought articles published in English between January 1990 and December 2025 using combinations of the following terms: “ventriculoperitoneal shunt,” “ventricular catheter,” “stereotactic,” “neuronavigation,” “electromagnetic navigation,” “image-guided,” “ultrasound-guided,” “catheter accuracy,” “proximal shunt failure,” and “hemorrhage.” Randomized controlled trials, comparative cohort studies, systematic reviews/meta-analyses, and large case series were eligible if they reported outcomes related to ventricular catheter accuracy, proximal failure, revision, or complications for guided versus conventional placement. Pediatric and adult studies were included. Studies focused exclusively on distal catheter techniques or non-shunt ventricular access were excluded unless they provided relevant mechanistic or stereotactic safety insights.

Reference lists of included studies were reviewed to identify additional relevant publications. Given heterogeneity in techniques (navigation, electromagnetic, ultrasound), populations, and outcome definitions, formal pooled meta-analysis was not performed; instead, we present a structured quantitative comparison of reported effect sizes and outcome directionality. The initial search was executed through December 2023 and was subsequently updated, using identical databases and search terms, through December 2025 prior to resubmission; this update identified two additional cohort analyses of intracranial hemorrhage following ventriculoperitoneal shunt surgery, which were incorporated into the hemorrhagic complications section.

### Epidemiology of hydrocephalus and shunt failure burden

In pediatric populations, hydrocephalus incidence is commonly reported in the range of approximately 0.5–0.8 per 1,000 live births, with prematurity-related intraventricular hemorrhage, congenital malformations, and aqueductal stenosis among common etiologies (1, 2). In adults, normal pressure hydrocephalus and secondary hydrocephalus due to trauma, hemorrhage, infection, or tumor represent major indications for shunting (2, 3).

Shunt failure imposes substantial clinical and societal burden. Classic health system analyses have emphasized that hydrocephalus treatment and shunting generate very high aggregate costs in the United States (16). Patient-level cost studies further show that the economic impact of shunt failure episodes varies by failure type, with infection-associated failures typically requiring longer hospitalization and multiple procedures, and obstruction often requiring focused revision (17). These realities underscore the potential value of interventions that reduce common failure modes, particularly proximal obstruction.

### Pathophysiology of proximal shunt failure

Proximal obstruction is primarily mechanical. Histopathological analyses have demonstrated catheter occlusion by choroid plexus tissue and ependymal ingrowth (6, 7). Fenestrations positioned partially within brain parenchyma are particularly susceptible to obstruction (6, 8). Ventricular wall contact has also been associated with increased malfunction rates (9).

Repeated catheter passes can increase cortical and subcortical trauma and may contribute to catheter tract hemorrhage and local inflammatory responses, plausibly increasing obstruction risk (8, 10). Observational data support that optimal intraventricular positioning correlates with improved proximal durability (8, 9).

### Technical rationale for stereotactic guidance

The primary advantage of stereotactic guidance is improved anatomical precision (8–12). Preoperative imaging enables individualized trajectory planning toward the frontal horn or atrium while avoiding ventricular walls and the choroid plexus (Figure 1). Frameless neuronavigation provides real-time trajectory visualization and depth control (8, 9). Electromagnetic systems provide similar targeting without rigid head fixation, offering practical advantages in pediatric populations (12, 13).

**Figure 1:**
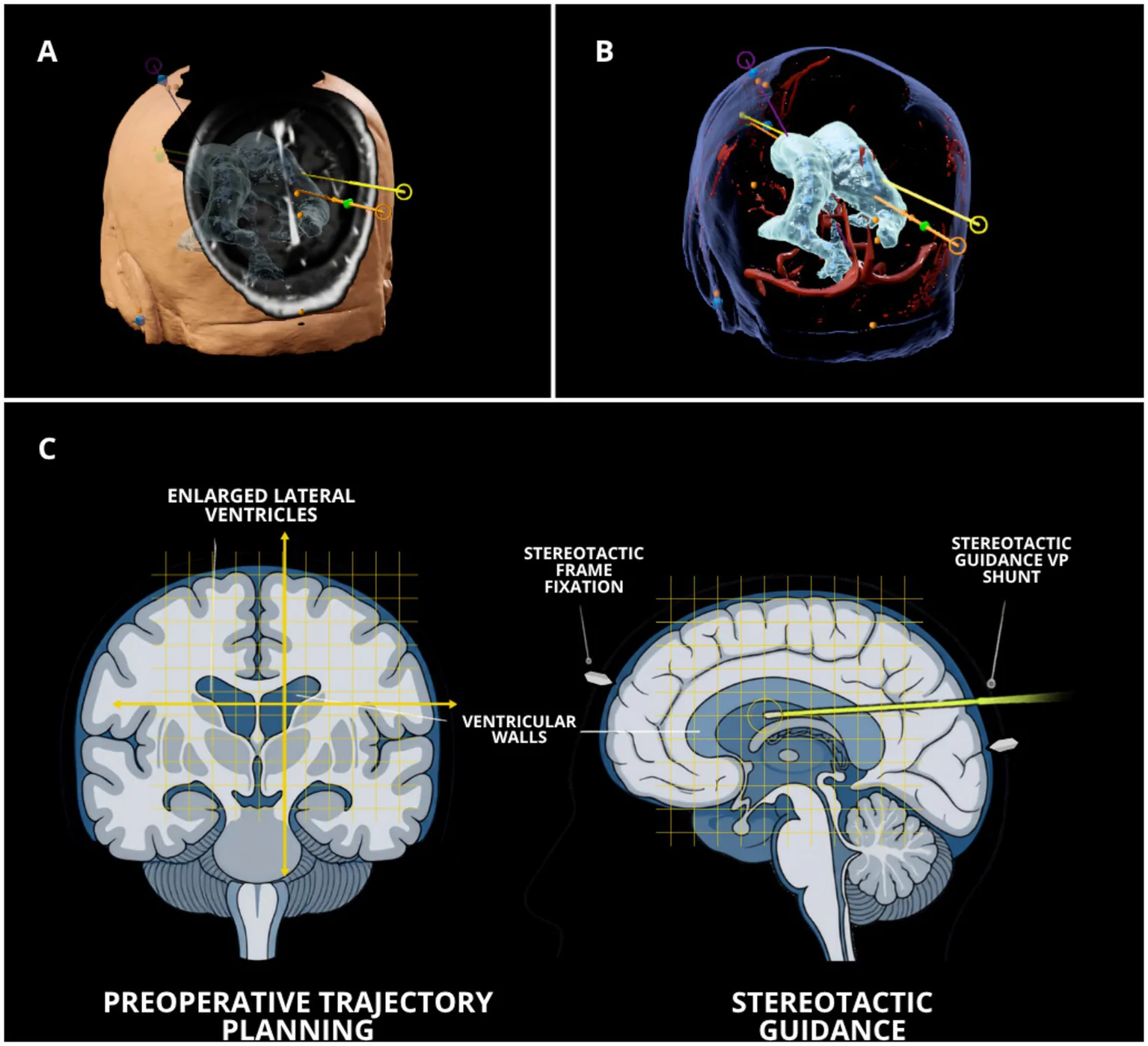
Stereotactic positioning of a ventricular catheter for VP shunt. Preoperative imaging and stereotactic guidance enhance anatomical precision, reproducibility, vascular structures avoidance and first-pass cannulation success during ventricular catheter placement. **(A)** shows an individualized three-dimensional cranial reconstruction (Elements Software, Brain Lab Inc., Munich, Germany) used for preoperative trajectory planning toward the target ventricular space, such as the frontal horn or atrium. **(B)** shows a three-dimensional neuronavigational rendering of the ventricular system (blue) and surrounding cerebral vasculature (red), demonstrating how trajectory planning allows for the deliberate avoidance of sulci, visible cortical vessels, and the choroid plexus to mitigate the risks of catheter tract hemorrhage and subsequent proximal mechanical occlusion. **(C)** shows anatomical and technical schematics illustrating a coronal brain cross-section overlaid with a stereotactic coordinate grid for target calculation (left), and a sagittal view depicting real-time guided catheter insertion utilizing stereotactic frame fixation (right). This guided approach provides accurate depth control and ensures complete intraventricular positioning of the perforated catheter holes, thereby preventing suboptimal ventricular wall contact or intraparenchymal fenestration placement.

Randomized and observational studies consistently demonstrate improved first-pass cannulation success with guided techniques compared to freehand placement (10, 14). Reduced cannulation attempts may reduce cortical injury and technical failure due to suboptimal positioning.

Trajectory planning also supports avoidance of visible vascular structures and sulci. Although the two procedures serve different populations, VP shunt and DBS placement share similar trajectory-related risks. Also, computer-assisted stereotactic approaches in other ventricular procedures demonstrate the feasibility and safety of precise trajectory planning (18). Although dedicated shunt trials powered for hemorrhage outcomes are lacking, improved first-pass success and planned avoidance of vascular-rich regions provide a plausible pathway to reduce bleeding risk. Table 1 summarizes the principal technical and outcome-related differences between freehand and stereotactic/image-guided ventricular catheter placement.

**Table 1:** Illustrative comparison of freehand versus stereotactic/image-guided ventricular catheter placement.

Domain	Freehand placement	Stereotactic/image-guided placement
Targeting	Surface landmarks, estimated angle/depth	Imaging-based target + real-time trajectory guidance
Accuracy (typical reported ranges)	Lower; non-ideal placement not uncommon	Higher; optimal placement more frequent
First-pass success	Variable; may require redirection	Higher; improved primary puncture success
Passes/brain penetrations	May increase in small/distorted ventricles	Typically reduced due to planned trajectory
Proximal obstruction risk	Higher if wall/choroid contact or intraparenchymal fenestrations	Potentially reduced via optimized tip positioning
Hemorrhage risk (mechanism)	Increased, with multiple passes/blind redirection	Potentially reduced via fewer passes + trajectory planning/avoidance
Best-use scenarios	Enlarged ventricles, straightforward anatomy	Small/slit ventricles, distorted anatomy, midline shift, revisions, complex trajectories
Workflow	Minimal setup	Registration/planning setup; equipment/training requirements

(Comparative interpretation supported by studies in Sections below (8–14).)

## Clinical evidence

### Randomized controlled evidence

The Guided Application of Ventricular Catheters (GAVCA) trial (14) represents the most rigorous multicenter, randomized, controlled evaluation of guided versus freehand ventricular catheter placement to date. Enrolling 139 patients in the primary intention-to-treat (ITT) analysis (70 guided using a mobile health application-assisted tool vs. 69 freehand), the study evaluated catheter placement across three distinct anatomical dimensions. While the primary endpoint of optimal grade I ipsilateral placement showed a positive trend that did not reach statistical significance in the ITT population [70.0% vs. 56.5%; odds ratio (OR) 1.79, 95% confidence interval (CI) 0.89–3.61; *p* = 0.099], guided placement achieved a statistically significant improvement in the primary successful puncture rate on the first attempt (100% vs. 91.3%; *p* = 0.012). Furthermore, complete intraventricular positioning of the multiperforated catheter holes was significantly higher in the guided group (81.4% vs. 65.2%; OR 2.34, 95% CI 1.07–5.1; *p* = 0.031).

Importantly, a *post hoc* analysis demonstrated that incorrect catheter placement (defined as >2 puncture attempts, extraventricular/partial positioning, or contralateral/third ventricle placement) was significantly reduced with guidance (10.0% vs. 31.9%; OR 4.21, 95% CI 1.66–10.68; *p* = 0.001). Despite these anatomical superiority metrics, early ventricular catheter revision rates did not significantly differ between groups (2 revisions in guided vs. 1 in freehand; *p* = 0.57).

As the trial authors noted, a 30-day timeframe is fundamentally insufficient to capture long-term proximal mechanical durability; delayed obstruction likely develops over time as progressive astrogliosis and inflammatory tissue responses around suboptimal perforation holes invade the intraluminal space. Consequently, longer-term follow-up studies specifically powered for revision-free catheter survival are required to determine whether precise initial perforation placement mitigates delayed proximal failure.

### Observational evidence

Retrospective cohort studies consistently report improved catheter accuracy with stereotactic or ultrasound-guided placement (8, 9). In parieto-occipital catheter placement, guided techniques were associated with improved freedom from proximal malfunction (9). Single-surgeon stereotactic series report very low proximal malfunction rates, supporting biological plausibility that improved tip position reduces mechanical failure (10). In adult quality improvement initiatives, integration of neuronavigation has been associated with improved shunt survival, although confounding by concurrent workflow changes cannot be excluded (19).

### Pediatric and small ventricle populations

Navigation may be especially valuable in slit ventricles, small ventricles, and pediatric hydrocephalus (11–13). Electromagnetic neuronavigation has demonstrated high feasibility and first-pass success in infants and children (12). Frameless stereotactic approaches have also been reported as useful in idiopathic intracranial hypertension, where ventricular size may be small and freehand cannulation difficult (20).

A systematic review/meta-analysis in pediatric hydrocephalus found improved radiographic accuracy with image guidance but no consistent reduction in overall revision rate, likely reflecting heterogeneity of techniques and the multifactorial nature of shunt failure (13). This distinction supports focusing future outcome studies on proximal malfunction rather than total revision.

### Idiopathic intracranial hypertension and normal-sized ventricles

Idiopathic intracranial hypertension (IIH) represents the clinical scenario in which stereotactic guidance is arguably most indispensable, because the ventricles are frequently normal-sized or slit-like and freehand cannulation is correspondingly unreliable. Maher et al. first demonstrated that stereotactically planned VP shunt placement was feasible and effective in refractory IIH, overcoming the longstanding reluctance to shunt normal or small ventricles (21). Woodworth et al. subsequently reported that frameless stereotactic ventricular shunts placed in IIH patients—despite the absence of ventriculomegaly—were safe and durable, with no revisions attributable to proximal catheter obstruction or shunt infection and sustained headache relief in approximately half of patients at two years (20). In a comparative analysis of neuroendoscopy, frameless stereotaxy, and intraoperative CT for IIH shunting, Yim et al. found that image-based techniques improved catheter placement accuracy and supported favorable proximal catheter outcomes (22).

Electromagnetic frameless neuronavigation has extended these advantages to the IIH population specifically, with Kandasamy et al. describing electromagnetic stereotactic VP cerebrospinal fluid shunting as a reliable means of achieving accurate proximal catheter placement in patients without dilated ventricles (23). The benefit also extends to children, in whom IIH is rarer and ventricular targets are especially demanding; Heyman et al. reported that neuronavigated VP shunt insertion was an effective treatment for medically resistant pediatric IIH, relieving headache and stabilizing vision (24). Collectively, these series establish IIH and other normal- or small-ventricle presentations as the contexts in which the accuracy gains quantified for image guidance (8) translate most directly into avoidance of failed proximal cannulation.

### Quantitative comparative evidence synthesis

Although heterogeneity limits formal pooling, available data allow structured quantitative interpretation (Table 2). Across comparative cohorts, optimal intraventricular catheter placement with freehand techniques is often reported around 60–70%, whereas guided techniques achieve approximately 80–95% in similar contexts, representing an absolute improvement in the range of 15–30% (8,9; Figure 2). In the GAVCA randomized trial, complete intraventricular perforation positioning was 81% with guidance versus 65% freehand (absolute difference 16%) (14).

**Table 2:** Structured summary of comparative studies of freehand vs. image-guided ventricular catheter placement.

Study	Design	N	Accuracy (Guided vs Freehand)	Proximal failure
Wilson et al. (8)	Retrospective cohort	249	~90% vs. ~ 65% optimal	Reduced in guided group
Wilson et al. (9)	Retrospective cohort	257	81% vs. 67% optimal	Improved proximal survival
Sampath et al. (10)	Series	70	100% single-pass (reported)	None observed
Thomale et al. (14)	RCT	139	81% vs. 65% optimal perforation placement	No early difference
Spennato et al. (13)	Systematic review/meta-analysis	2017	Improved radiographic accuracy	No pooled reduction

Study quality and risk of bias vary substantially across the entries. Only Thomale et al. (14) is a randomized controlled trial; the remaining comparative entries are retrospective, non-randomized cohorts or single-arm series subject to selection bias, unblinded outcome adjudication, heterogeneous definitions of optimal catheter position, and variable follow-up duration. Values are therefore presented for directional comparison rather than as pooled or statistically comparable estimates, and cross-study differences should not be interpreted as head-to-head effect sizes. N denotes the number of patients in each individual cohort or series, except for Spennato et al. (13), where N reflects the pooled sample aggregated across all studies included in that systematic review and meta-analysis and is therefore not a single-cohort sample size directly comparable to the other rows.

**Figure 2:**
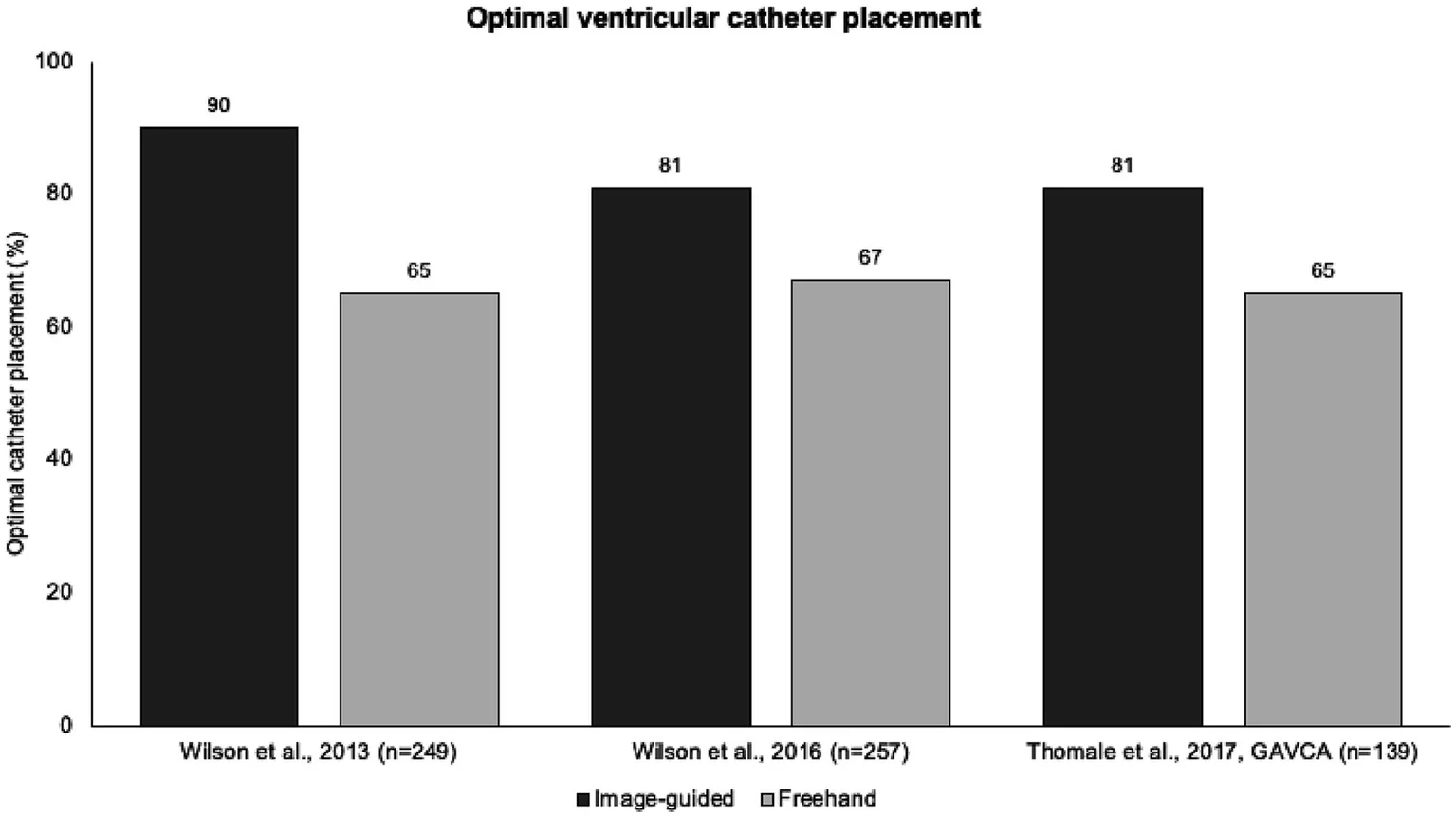
Comparative rates of optimal ventricular catheter placement with image-guided versus freehand technique, as reported in the three comparative studies summarized in Table 2 (8, 9, 14). Bars represent the proportion of catheters achieving optimal or complete intraventricular positioning. Values are drawn from separate study populations with differing designs and positioning definitions and are displayed for directional comparison only; they do not constitute pooled or statistically comparable estimates.

Regarding proximal malfunction, observational studies suggest that improved positioning is associated with improved proximal survival (9). While pooled hazard ratios cannot be derived consistently due to variable reporting, directionality across comparative studies supports a probable reduction in proximal obstruction when placement is optimized (8–10). The pediatric meta-analysis found no uniform reduction in overall revision rate, underscoring that infection and distal complications can dilute the effect of proximal accuracy when composite endpoints are used (13).

### Hemorrhagic complications: incidence, risk factors, and translational lessons from DBS trajectory planning

Intracranial hemorrhage following VP shunt surgery is generally considered uncommon but clinically significant. In a large contemporary VP shunt cohort with routine postoperative CT imaging, intracranial hemorrhage (including tract-related hematoma and intraventricular hemorrhage) occurred in approximately 5.85% of patients (25). Risk factors included perioperative antiplatelet/anticoagulant exposure and technical/anatomical variables, and the authors emphasized that surgical planning should prioritize vascular anatomy (25). Other clinical series and analyses emphasize that increased postoperative imaging can improve detection of minor or asymptomatic bleeding (26).

Repeated cannulation attempts represent a plausible procedural risk factor for tract bleeding, particularly in small ventricles and distorted anatomy, where freehand placement may require redirection (8, 10). Stereotactic guidance may mitigate hemorrhage risk through two complementary mechanisms: improved first-pass success, or less repeated penetrations (14); and preoperative planned trajectory to avoid sulci, cortical veins, and vascular-rich regions such as the choroid plexus (18, 25).

A useful translational comparison comes from deep brain stimulation (DBS) surgery, where decades of stereotactic evolution have increasingly emphasized trajectory planning and vascular/sulcal avoidance. A recent DBS meta-analysis reported intracranial bleeding incidence of approximately 2.5% per patient (and 1.4% per lead), reflecting the aggregate of multiple eras and techniques (27). Importantly, a large contemporary DBS series highlights the strong association between sulcal proximity/penetration and vascular complications, reporting markedly higher complication rates with sulcal traversal compared with trajectories clearly positioned within a gyrus, reinforcing the value of sulcal avoidance (28). The same DBS literature emphasizes that deliberate trajectory planning paradigms can reduce hemorrhagic risk (28). While VP shunt catheter placement and DBS differ in target and procedural details, both share trans-cortical cannulation toward deep structures, supporting the plausibility that trajectory planning principles are transferable.

No randomized shunt trial has been powered primarily for hemorrhage reduction; therefore, the hemorrhage benefit of stereotaxy in shunt surgery remains a hypothesis supported by mechanism, first-pass success improvements, and translational stereotactic experience (14, 27, 28). Prospective shunt studies with standardized postoperative imaging and hemorrhage grading are needed to quantify any true reduction attributable to guidance.

### Health economic considerations and preliminary cost-effectiveness modeling

Shunt revision imposes substantial healthcare costs, including operative expenses, hospitalization, imaging, and caregiver burden. A classic analysis described the large aggregate national cost burden attributable to shunt treatment in the United States (16). Patient-level economic studies further demonstrate that the cost of shunt failure episodes varies by failure type, with infection generally far more costly than obstruction due to prolonged hospitalization and multi-stage management (17). Cost analyses of shunt durability and revisions also emphasize the financial significance of revisions over time (29).

Cost-effectiveness of stereotactic guidance depends on whether improved catheter accuracy translates into fewer proximal obstructions and revisions. A simplified decision-analytic framework can be applied. If baseline proximal revision risk is 25% in a high-risk group (e.g., small ventricles, revisions) and navigation yields an absolute reduction of 5%, the number needed to treat (NNT) to prevent one proximal revision would be 20; if absolute reduction is 10%, NNT decreases to 10. Given the substantial direct costs and indirect burdens associated with revision episodes (16, 17, 29), preventing even a small number of revisions annually may offset navigation-associated costs in high-volume centers, particularly where navigation infrastructure already exists.

However, formal cost-utility analyses incorporating quality-adjusted life years (QALYs), indirect costs, and sensitivity analyses are limited in this domain. Future prospective studies should include economic endpoints alongside proximal durability outcomes to clarify real-world value.

## Limitations

The available literature is predominantly retrospective with heterogeneity in patient selection, ventricular size, surgical approach, navigation modality, and follow-up duration. Many studies use radiographic positioning or overall revision as endpoints, limiting precision in estimating the effect on isolated proximal malfunction. Surgeon experience and learning curve effects are rarely controlled for, introducing potential performance bias. Additionally, navigation may be preferentially used in anatomically challenging cases, creating selection bias that can either inflate or underestimate apparent benefit. No large randomized trial has been powered to evaluate long-term proximal survival or hemorrhage reduction as primary endpoints. Economic modeling remains preliminary and formal cost-utility analyses are sparse. Consequently, while accuracy benefit is well supported, definitive quantification of long-term survival, hemorrhage reduction, and cost-effectiveness requires further prospective investigation.

## Future directions

Future studies should prioritize (i) long-term proximal survival endpoints rather than composite revision rates, (ii) stratification by ventricular size and revision status, (iii) standardized radiographic grading systems for catheter position and hemorrhage, (iv) incorporation of vascular imaging into planning where feasible, and (v) health economic analyses including QALYs and sensitivity modeling. Pragmatic trials or stepped-wedge implementations may be particularly informative for institutional adoption questions.

## Conclusion

Stereotactic guidance improves anatomical accuracy and first-pass success in ventricular catheter placement. Observational evidence suggests reduced proximal malfunction in selected populations, although overall revision rates remain influenced by infection, distal failure, and valve-related complications. Hemorrhage after VP shunt placement is uncommon but important; emerging VP shunt data and translational experience from DBS trajectory planning support the hypothesis that planned trajectories and vascular/sulcal avoidance may reduce bleeding risk. High-quality stratified randomized trials and prospective economic evaluations are required to define long-term clinical and cost-effectiveness benefits.
